# Complementary feeding practices among rural Bangladeshi mothers: Results from WASH Benefits study

**DOI:** 10.1111/mcn.12654

**Published:** 2018-08-13

**Authors:** Kaniz Jannat, Stephen P. Luby, Leanne Unicomb, Mahbubur Rahman, Peter J. Winch, Sarker M. Parvez, Kishor K. Das, Elli Leontsini, Pavani K. Ram, Christine P. Stewart

**Affiliations:** ^1^ Environmental Intervention Unit, Infectious Disease Division International Centre for Diarrhoeal Disease Research Dhaka Bangladesh; ^2^ Medicine ‐ Med/Infectious Diseases Stanford University Stanford California; ^3^ Department of International Health Johns Hopkins Bloomberg School of Public Health Baltimore Maryland; ^4^ Department of Epidemiology and Environmental Health University at Buffalo Buffalo New York; ^5^ Department of Nutrition University of California Davis Davis California

## Abstract

Inappropriate complementary feeding contributes to linear growth faltering in early childhood. Behaviour change interventions have been effective at improving practice, but few studies have investigated the effects of multicomponent integrated interventions. We conducted a cluster‐randomized controlled trial in rural Bangladesh in which geographic clusters were randomized into seven arms: water treatment (W), sanitation (S), handwashing (H), water, sanitation, and handwashing (WSH), improved nutrition with infant and young child feeding messages and lipid‐based nutrient supplementation for 6‐ to 24‐month olds (N), N+WSH, and control. The objective of this paper was to examine the independent and combined effects of interventions on indicators of complementary feeding. Approximately 1 and 2 years after initiation of the intervention, research assistants surveyed mothers about infant feeding practices. Complementary feeding was examined using the World Health Organization indicators of infant and young child feeding practices. We used Poisson regression models to estimate prevalence ratios and linear regression models for prevalence differences with clustered sandwich estimators to adjust for clustering. A total of 4,718 households from 720 clusters were surveyed at year 1 and 4,667 at year 2. The children in the nutrition arms had a higher prevalence of meeting the minimum dietary diversity score compared with controls (year 1: N: 66.4%; N+WSH: 65.0% vs. C:32.4%; year 2: N: 91.5%; N+WSH: 91.6% vs. C:77.7%). Children in the nutrition arms received diverse food earlier than the children in control arm. In addition, the average consumption of lipid‐based nutrient supplementation was >90% in each follow‐up. Nutrition‐specific interventions could be integrated with nutrition‐sensitive interventions such as WSH without compromising the uptake of the nutrition intervention.

**Key messages**

- The promotion of optimal infant and young child feeding practices was associated with an earlier introduction of nutrient‐dense complementary foods and a greater likelihood of meeting the minimum dietary diversity and minimum acceptable diet scores.
- Provision of lipid‐based nutrient supplements to infants 6–24 months of age did not displace nutrient‐dense complementary foods.
- Nutrition‐specific interventions could be integrated with nutrition‐sensitive interventions such as water, sanitation, and handwashing with soap promotion without compromising the uptake of the nutrition intervention.

## 1 INTRODUCTION

Child undernutrition contributes substantially to child mortality and morbidity in low‐income countries. In 2011, the cumulative effect of undernutrition, including fetal growth restriction, inadequate breastfeeding, stunting, wasting, and deficiencies of vitamin A and zinc was estimated to cause 3.1 million child deaths (45% of all child deaths) among those younger than 5 years of age (Black et al., 2013). Effects of early growth faltering on various life consequences such as adverse health outcome, lower educational and economic attainments have been identified through a number of studies (Dewey & Begum, 2011). Diet quality among young children is often poor, characterized by low dietary diversity and inadequate consumption of nutrient‐dense foods. A study across 21 low‐income countries found that inadequate complementary feeding practices were associated with a negative growth pattern (Onyango, Borghi, de Onis, del Carmen Casanovas, & Garza, 2014). In particular, the dietary diversity score was consistently associated with attained length. Interventions aimed at improving complementary feeding practices have been associated with modest improvements in child growth (Dewey & Adu‐Afarwuah, 2008).

It is possible that interventions have had only a limited impact on growth because they have not addressed underlying determinants of growth faltering. For this reason, there has been a greater interest on integration of nutrition‐sensitive interventions such as WASH with nutrition‐specific interventions (Bhutta et al., 2013; Ruel & Alderman, 2013). Integration of health services is extensively promoted with a view of greater efficiency and improved health outcomes (Reynolds & Sutherland, 2013). However, the benefits of integrated programmes are highly debated (Atun, de Jongh, Secci, Ohiri, & Adeyi, 2009). Staff or supervisors may become overloaded; participants might become overwhelmed, unable to act on all of the recommendations provided; and activities need to be well coordinated across domains (DiGirolamo, Stansbery, & Lung'aho, 2014). Nevertheless, there is some evidence that nutrition and child cognitive development interventions obtained better results when they were integrated (Black, Pérez‐Escamilla, & Fernandez Rao, 2015; Vazir et al., 2013).

We hypothesized that a nutrition behaviour change intervention would improve complementary feeding practices and that an integrated nutrition, water, sanitation, and handwashing intervention would equally be effective at improving complementary feeding practices. Our objective for this paper was to examine the independent and combined effects of interventions focused on improvements in nutrition, water, sanitation, and handwashing on indicators of complementary feeding (dietary diversity, meal frequency, and overall dietary adequacy).

## 2 METHODS

### 2.1 Study design

**Figure 1:**
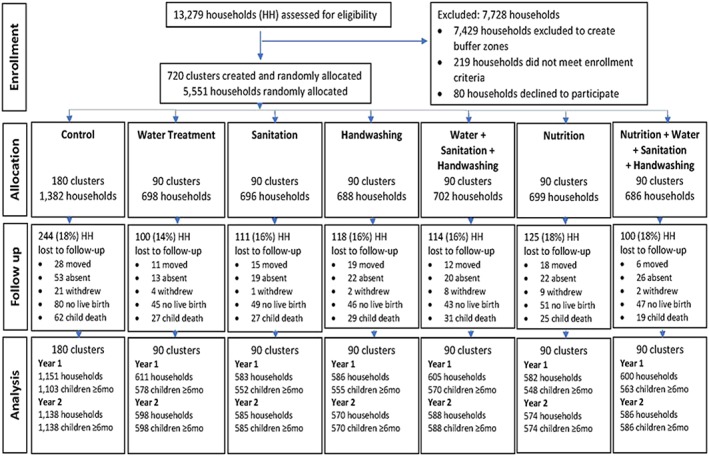
Summary of participant enrolment, randomization, retention, and analysis for infant food frequency

The WASH Benefits Bangladesh study (http://clinicaltrials.gov identifier: NCC01590095) was a community‐based cluster‐randomized control trial conducted in rural Gazipur, Tangail, Mymensingh, and Kishoreganj districts of central Bangladesh. These sites were selected according to their water characteristics and absence of any major ongoing or upcoming water, sanitation, handwashing, or nutrition intervention by the Government of Bangladesh or other non‐government organizations. In rural Bangladesh, households are usually organized into compounds that share a common latrine, water source, and often kitchen. Research assistants visited the compounds and identified pregnant women in their first or second trimesters and their newborn children became the index children for the study. A total of 5,551 pregnant women (households) were enrolled in the study (Figure 1). Using the global positioning system (ArcGIS mapping software) coordinates, the eight nearest pregnant women formed a cluster. We randomized 720 clusters in blocks of eight clusters, with 5,551 pregnant women, into seven study arms: single water (W), sanitation (S), handwashing with soap (H), and nutrition (N) intervention arms and combined water, sanitation, and handwashing (WSH), nutrition and WSH intervention arms (N+WSH), and a double sized control arm (C) in which there were no intervention activities. Due to the nature of the intervention, neither the participants nor the data collectors were masked. The detail of the study design and rationale have been published (Arnold et al., 2013; Luby et al., 2018).

### 2.2 Intervention design and delivery

Before starting the main trial, we conducted extensive formative research to identify the appropriate enabling technologies and behavioural intervention packages (Hulland et al., 2013; Hussain et al., 2017). We utilized the Integrated Behavioural Model for Water Sanitation and Hygiene (Dreibelbis et al., 2013) to develop the intervention content for each arm. We piloted and revised the interventions in the target populations over a 2‐year period prior to the main trial.

Community Health Workers (CHW) were recruited from the local community with an educational attainment of at least grade 8. We arranged rigorous trainings for the CHWs for each of the intervention components. The CHWs were closely monitored and supervised to adapt innovative approaches to evolving conditions at the field. Each CHW was responsible for one cluster comprising 6–8 eligible mothers. A cluster diameter was 1 km on average, ranging from 0.2 to 2.2 km. A buffer zone of 1 km was maintained between adjacent clusters. The CHWs were asked to conduct individual household meetings twice a month to deliver arm specific behaviour change messages. However, during the first 6 months of intervention, the visits were more frequent, at least four times a month.

The combined interventions required an approximate 3‐month period to roll out the different components successively. The interventions comprised both behaviour change components and hardware to support an enabling environment. In the arms that received the water interventions (W, WSH, and N+WSH), households were provided with an insulated drinking water storage container along with a regular supply of chlorine water treatment products (Aquatabs^TM^, Medentech, Wexford, Ireland). CHWs encouraged participants to store and drink safe water. The sanitation intervention recipient households (S, WSH, N+WSH) received instruments for safe disposal of child faeces and an improved double pit pour‐flush latrine. The behavioural objectives included the safe disposal of child faeces and the usage of improved latrines. To facilitate handwashing with soap after toilet use and before contact with food, households from the handwashing intervention arms (H, WSH, N+WSH) received two handwashing stations, one at the latrine and another at the cooking area. Handwashing stations included a water container with tap and basin and a soapy‐water storage bottle. Regular supplies of laundry detergent sachets were provided by the CHWs for preparing soapy water. Soapy water is a solution of detergent and water, an inexpensive alternative to bar soap (Amin et al., 2014; Ashraf et al., 2017). CHWs in H, WSH combined, and N+WSH combined arms provided intensive counselling on washing hands with soap at key times: before preparing food, before eating or feeding a child, after defecation, and after cleaning a child who had defected.

The nutrition intervention households (N and N+WSH) received information on maternal and child nutrition along with a monthly supply of small quantity (20 g/daily) lipid‐based nutrient supplements (LNS; Nutriset, Malaunay, France). CHWs tailored their nutrition recommendations according to the stages of pregnancy and child age. During pregnancy, they encouraged maternal dietary diversity and regular antenatal visits. Beginning in the last trimester of pregnancy, CHWs communicated about early initiation of breastfeeding, encouraged exclusive breastfeeding up to 6 months of age, and continued breastfeeding up to 2 years. CHWs conducted special sessions for mothers experiencing any challenges. Timely introduction (at 6 months of age) of complementary feeding and LNS were encouraged through a ritual and celebration of the infant's 6‐month birthday known as “*Mukhe Bhat*” (occasion of child eating rice for the first time). CHWs encouraged mothers to feed diverse family foods using illustrative flip charts. They also distributed sachets of LNS to the index children from 6 to 24 months of age. Families were provided with monthly rations of LNS enabling them to feed the index children two 10 g sachets per day. Mothers were encouraged to feed both sachets to the index child and to not share the LNS with their other children. CHWs placed reminder cue cards in the households illustrating food diversity. Children stopped receiving LNS when they reached 24 months of age. However, all other complementary feeding messaging continued until the year 2 survey round was complete. Flip charts used to deliver nutrition information and CHW implementation guide are available online (https://osf.io/x9fv7).

### 2.3 Data collection

At the baseline survey, research assistants collected information about demographic characteristics, current sanitation, and hygiene practices. We adapted the infant food frequency questionnaire from the World Health Organization (WHO) indicators of infant and young child feeding guideline (WHO, 2010). The first follow‐up visit occurred approximately 1.5 years after initiation of the intervention activities and an infant food frequency questionnaire (24‐hr recall and 7‐day recall) was administered. The second follow‐up survey occurred 1 year after the first, about 2.5 years after the initiation of intervention activities.

### 2.4 Data analysis

We grouped complementary foods into seven categories: grains, roots and tubers; legumes and nuts; dairy products; flesh foods; eggs; vitamin‐A rich fruits and vegetables; and other fruits and vegetables. LNS was not included in the food‐group categories for this analysis. We used the WHO indicators of infant and young child feeding guidelines to calculate minimum dietary diversity (MDD), minimum meal frequency (MMF), and minimum acceptable diet (MAD) scores (WHO, 2010). The MDD score indicates the proportion of children who received foods from four or more food categories on the previous day. MMF measures the proportion of children who received solid, semi‐solid, or soft foods the minimum numbers of time or more on the previous day. The minimum acceptable number is two or more times between the age of 6 and 8 months and three or more times thereafter. The MAD score is the proportion of children who met both the MDD and MMF scores. The proportion of children in each arm who met the MDD, MMF, or MAD scores were calculated for each survey round. Because all dietary indicators were dichotomous variables, our parameter of interest was the prevalence ratio (similar to RR = risk ratio). In addition, we calculated the proportion of children who received foods from four or more food categories and consumed animal source food all days in the past 7 days. We used the same food group categories for the 7‐day recall as described above. We do not present analysis for nonbreastfed children because they were few in number during both follow‐up points (85 at year 1 and 281 at year 2 follow‐up), and the guidelines for assessment is different. The WHO definitions typically pertain to children 6–24 months of age; however, in our study, we have chosen to include all children surveyed during year 2, approximately 80% of who ranged in age from 6 to 24 months. Household food insecurity was measured and analysed using Household Food Insecurity Access Scale and Household Hunger Scale indicator guide recommended by the Food and Nutrition Technical Assistance III Project (Ballard, Coates, Swindale, & Deitchler, 2011).

With the binary indicator variables of MDD, MMF, and MAD, we compared each intervention group with the control group in two ways. Because the prevalence of these outcomes was not rare, we used robust Poisson regression models (Petersen & Deddens, 2008) to estimate prevalence ratios (PR) along with their 95% confidence intervals. We also estimated prevalence differences between groups using linear regression models. For estimating the standard errors and confidence intervals of the estimates of prevalence, we used robust sandwich estimators to adjust for clustering effects due to the randomization blocks and clusters. Additionally, we repeated these analyses comparing the WSH+N with the N group.

The study was a cluster‐randomized trial and therefore the possibility of confounding was minimal. Nevertheless, we performed a regression analysis including additional covariates such as sex of the index child, parents' education, fathers' occupation, household income, geographical location, and seasonality (Table S2). We did not find any substantive change in the coefficients or confidence intervals and have not reported them in this paper.

### 2.5 Ethical considerations

Research assistants explained the study procedures to the household members. Research assistants then sought written informed consent from the head of the households, the pregnant women, and guardians of children less than 36 months of age prior to random assignment. The study was reviewed and approved by human subjects review committees at the icddr,b, Stanford University and University of California Berkeley.

## 3 RESULTS

The study arms were demographically similar at baseline (Table 1). More than 60% of the households had a total income less than 10,000 taka, approximately equivalent to 125 USD per month. The average household size was 4.7 (*SD* 2.2) members. Food insecurity was reported by approximately 30% of the households. Agricultural work, service, or business were the most common occupations among the fathers. About half of the mothers had more than primary level education.

**Table 1:** Characteristics of the study population at baseline and children at follow‐up

Indicators	Control^a^	Water	Sanitation	Handwashing	WSH	Nutrition	N+WSH
	*N* = 1,382% [95% CI]	*N* = 698% [95% CI]	*N* = 696% [95% CI]	*N* = 688% [95% CI]	*N* = 702% [95% CI]	*N* = 699% [95% CI]	*N* = 686% [95% CI]
Household characteristics							
Income <10,000 taka^b^	70 [67, 72]	67 [63, 70]	68 [65, 72]	71 [68, 74]	67 [63, 70]	66 [63, 70]	68 [64, 71]
Ownership of land	98 [98, 99]	99 [98, 99]	99 [98, 100]	98 [97, 99]	97 [96, 98]	98 [97, 99]	98 [97, 99]
Raise livestock	85 [83, 87]	85 [82, 87]	87 [84, 89]	87 [84, 90]	83 [79, 85]	86 [83, 88]	83 [80, 86]
Household size (mean, *SD*)	4.7 (2.3)	4.6 (2.2)	4.7 (2.1)	4.7 (2.2)	4.7 (2.1)	4.7 (2.2)	4.7 (2.1)
Food insecurity	33 [30, 35]	29 [26, 33]	32 [28, 35]	31 [28, 35]	31 [28, 35]	31 [28, 35]	29 [26, 33]
Paternal characteristics							
Occupation of the father							
Agriculture	30 [28, 32]	32 [29, 36]	29 [26, 33]	36 [33, 40]	31 [27, 34]	33 [30, 37]	30 [27, 34]
Service or business	30 [27, 32]	27 [24, 30]	30 [27, 34]	28 [24, 31]	31 [28, 34]	31 [27, 34]	31 [28, 35]
Working abroad	7 [5, 8]	7 [5, 9]	7 [5, 9]	5 [3, 7]	6 [4, 8]	6 [4, 8]	5 [4, 7]
Other	34 [31, 36]	35 [31, 38]	34 [31, 38]	31 [28, 35]	32 [29, 36]	30 [27, 34]	34 [30, 37]
Education, more than primary	41 [38, 43]	40 [36, 43]	41 [37, 44]	37 [34, 41]	40 [37, 44]	40 [36, 43]	37 [34, 41]
Maternal characteristics							
Education, more than primary	53 [51, 56]	54 [50, 58]	52 [48, 56]	53 [49, 57]	54 [50, 58]	54 [50, 57]	50 [46, 54]
Height <145 cm	12 [10, 14]	14 [11, 17]	14 [12, 17]	13 [10, 15]	13 [11, 16]	14 [11, 17]	15 [12, 18]
Diet in past 7 days (≥1 day)							
Meat, fish, or eggs	99 [99, 100]	100	100	100	99 [99, 100]	99 [98, 100]	99 [99, 100]
Dairy	53 [50, 55]	54 [50, 58]	54 [51, 58]	54 [50, 58]	51 [48, 55]	50 [46, 54]	55 [51, 58]
Vitamin A rich (yellow) fruits or veg.	47 [45, 50]	48 [44, 52]	48 [44, 52]	47 [43, 50]	48 [44, 51]	50 [46, 53]	50 [46, 54]
Child characteristics at follow‐up							
Sex (male) at year 1	49 [46, 52]	50 [46, 54]	51 [47, 55]	50 [46, 55]	52 [48, 56]	51 [47, 55]	47 [43, 51]
Sex (male) at year 2	50 [47, 53]	50 [46, 54]	51 [46, 55]	49 [45, 53]	52 [48, 56]	51 [47, 55]	46 [42, 50]
Age (months) at year 1 (mean, *SD*)	8.8 (1.7)	8.8 (1.7)	8.8 (1.7)	8.8 (1.7)	8.7 (1.8)	8.6 (1.7)	8.7 (1.7)
Age (months) at year 2 (mean, *SD*)	22.4 (2.0)	22.5 (2.0)	22.5 (2.0)	22.5 (2.1)	22.4 (2.1)	22.4 (2.1)	22.4 (2.0)

a None of the indicators were significantly different compared with the control arm.

b 1 USD = 80 taka.

At the year 1 follow‐up visit, 4,747 caregivers (85.5%) provided information on infant feeding practices for the index children and at the year 2 follow‐up, 4,667 (84.1%) provided this information. The average age of the children at the year 1 follow‐up was around 9 months and 22 months at year 2. Almost all the children (98%) were breastfeeding during the year 1 follow‐up that remained equally high (90%) during year 2 (Table 1). In the nutrition arms, the reported average consumption of LNS among children 6–24 months was ~93% in year 1 and year 2 follow‐up (Luby et al., 2018). At the year 2 follow‐up point, 20% of children had aged out of eligibility and were no longer receiving LNS.

At year 1, the proportion of mothers reporting consumption of grains, legumes and nuts, flesh foods, eggs, vitamin A‐rich fruits and vegetables, and other fruits and vegetables by their children were significantly higher in the two nutrition arms than in the control arm (Table 2). Similarly, at year 2, reported consumption of legumes and nuts, dairy products, flesh foods, eggs, vitamin A‐rich fruits and vegetables, and other fruits and vegetables were significantly higher in the nutrition arms compared with the controls. However, the consumption of all food categories had increased across all arms over time and so the magnitude of the difference between the nutrition arms and the control arm in year 2 was not as large as at the year 1 visit. Consumption–prevalence of foods from the seven categories was similar among children from the non‐nutrition intervention arms (W, S, H, and WSH) and the control arm at both time points.

**Table 2:** Reported prevalence of infant and young child feeding in the past 24 hr after 1 and 2 years of intervention

Food groups consumed	Control	Water	Sanitation	Handwashing	WSH	Nutrition	N+WSH
	*N* = 1,103% [95% CI]	*N* = 578% [95% CI]	*N* = 552% [95% CI]	*N* = 555% [95% CI]	*N* = 570% [95% CI]	*N* = 548% [95% CI]	*N* = 563% [95% CI]
Year 1							
Grains	91 [90, 93]	93 [91, 95]	94 [92, 96]*	93 [91, 95]	92 [90, 94]	95 [93, 97]*	98 [96, 99]*
Legumes and nuts	26 [24, 29]	28 [24, 32]	30 [26, 34]	29 [25, 33]	28 [25, 32]	45 [40, 49]*	49 [45, 54]*
Dairy products	35 [32, 38]	37 [33, 41]	38 [34, 42]	37 [33, 41]	36 [33, 41]	34 [30, 38]	32 [28, 36]
Flesh foods	25 [23, 28]	29 [25, 32]	24 [21, 28]	30 [26, 34]	27 [23, 31]	57 [52, 61]*	57 [53, 61]*
Eggs	18 [16, 21]	24 [20, 27]*	24 [21, 28]*	24 [20, 28]*	17 [14, 20]	39 [35, 43]*	33 [29, 37]*
Vit‐A rich fruits and veg.	23 [21, 26]	24 [21, 28]	26 [23, 30]	29 [26, 33]*	28 [24, 32]*	53 [49, 57]*	49 [45, 53]*
Other fruits and veg.	56 [53, 59]	59 [55, 63]	59 [55, 63]	60 [55, 64]	63 [59, 67]*	80 [77, 84]*	81 [78, 84]*

	*N* = 1,138% [95% CI]	*N* = 598% [95% CI]	*N* = 585% [95% CI]	*N* = 570% [95% CI]	*N* = 588% [95% CI]	*N* = 574% [95% CI]	*N* = 586% [95% CI]
Year 2							
Grains	100	99 [98, 100]	100	99 [98, 100]	100	100	100
Legumes and nuts	44 [41, 47]	43 [39, 47]	47 [43, 51]	46 [42, 50]	41 [37, 45]	53 [49, 58]*	53 [49, 57]*
Dairy products	40 [38, 43]	41 [37, 46]	42 [38, 46]	42 [38, 46]	42 [38, 46]	46 [42, 50]*	49 [45, 53]*
Flesh foods	75 [73, 78]	78 [75, 82]	77 [74, 81]	77 [74, 81]	79 [75, 82]	85 [82, 88]*	86 [83, 89]*
Eggs	37 [34, 40]	39 [35, 43]	42 [38, 46]*	38 [34, 42]	41 [37, 45]	48 [44, 52]*	52 [48, 56]*
Vit‐A rich fruits and veg.	42 [39, 45]	47 [43, 51]*	46 [42, 51]	51 [47, 55]*	45 [41, 49]	59 [55, 63]*	61 [57, 65]*
Other fruits and veg.	97 [95, 97]	97 [96, 99]	97 [95, 98]	97 [95, 98]	98 [96, 99]	99 [98, 100]*	99 [97, 99]*

* *P* value < 0.05. Each intervention arm was compared with the control arm.

Children in the nutrition intervention arms were more likely to meet the MDD score in both of the follow‐up surveys compared with those in the control arm (year 1: N: 66.4%; N+WSH: 65.0% vs. C:32.4%; year 2: N: 91.5%; N+WSH: 91.6% vs. C:77.7%; Table 3). This equated to a twofold greater prevalence of meeting the MDD score at year 1, but only a 10% greater prevalence at year 2. MMF was achieved by more than 80% of the children in the year 1 follow‐up and by the year 2 follow‐up, it was 100% across all the arms. Thus, there were no measurable differences in achieving MMF at year 2 follow‐up (PD = 0, PR = 1). Children were more likely to receive a MAD in the nutrition intervention arms than the control arm in both the follow‐up surveys (year 1: N: 65.2%; N+WSH: 63.8% vs. C:30.7%; year 2: N: 90.4%; N+WSH: 91.1% vs. C:77.0%; Table 3). None of the indicators were statistically more prevalent in the integrated N+WSH versus the single nutrition intervention arms (Table S1). MDD score and MAD score were also similar in the non‐nutrition intervention arms (W, S, H, and WSH) and controls.

**Table 3:** Effect of the intervention on infant and young child feeding practices comparing each intervention arm with the control arm

	Year 1				Year 2			
	*N*	%	PD^a^ [95% CI]	PR [95% CI]	*N*	%	PD^a^ [95% CI]	PR [95% CI]
Minimum dietary diversity								
Control	1,103	32.4	Ref	Ref	1,138	77.7	Ref	Ref
Water	578	36.0	3.1 [−1.8, 8.0]	1.1 [1, 1.3]	598	80.8	2.9 [−1.1, 6.8]	1.0 [1.0, 1,1]
Sanitation	552	37.1	4.8 [0, 9.4]	1.1 [1, 1.3]	585	82.0	3.9 [0.2, 7.7]	1.1 [1.0, 1.1]
Handwashing	555	40.4	7.2 [2.5, 12.0]*	1.2 [1.1, 1.4]*	570	81.1	3.2 [−0.5, 7.0]	1.0 [1.0, 1.1]
WSH	570	36.5	3.7 [−0.5, 8.0]	1.1 [1, 1.3]	588	80.3	2.4 [−1.3, 6.1]	1.0 [1.0, 1.1]
Nutrition	548	66.4	33.9 [29.0, 39.0]*	2.0 [1.8, 2.3]*	574	91.5	13.7 [10.3, 17.1]*	1.2 [1.1, 1.2]*
N+WSH	563	65.0	32.8 [27.8, 37.8]*	2.0 [1.8, 2.3]*	586	91.6	13.9 [10.5, 17.2]*	1.2 [1.1, 1.2]*
Minimum meal frequency								
Control	1,074	85.0	Ref	Ref	1,020	100	—b	—b
Water	563	86.3	1.4 [−2.1, 5.0]	1.0 [1.0, 1.1]	532	100	—b	—b
Sanitation	539	87.8	3.0 [−0.3, 6.3]	1.0 [1.0, 1.1]	538	100	—b	—b
Handwashing	549	85.4	0.4 [−3.2, 4.1]	1.0 [0.9, 1.0]	518	100	—b	—b
WSH	553	88.3	3.5 [−0.1, 7.0]	1.0 [1.0, 1.1]	529	100	—b	—b
Nutrition	540	93.5	8.8 [5.7, 12.0]	1.1 [1.1, 1.1]	530	100	—b	—b
N+WSH	555	95.0	10.1 [7.1, 13.1]	1.1 [1.1, 1.2]	517	99.8	—b	—b
Minimum acceptable diet								
Control	1,074	30.7	Ref	Ref	1,020	77.3	Ref	Ref
Water	563	34.5	3.3 [−1.6, 8.1]	1.1 [1.0, 1.3]	532	80.1	2.7 [−1.6, 7.0]	1.0 [1.0, 1.1]
Sanitation	539	35.3	4.5 [−0.1, 9.1]	1.1 [1.0, 1.3]	538	81.0	3.6 [−0.5, 7.6]	1.0 [1.0, 1.1]
Handwashing	549	39.0	7.4 [2.7, 12.1]*	1.2 [1.1, 1.4]*	518	81.3	4.0 [0, 7.9]	1.1 [1.0, 1.1]
WSH	553	35.1	4.0 [−0.3, 8.1]	1.1 [1.0, 1.3]	529	80.3	3.0 [−1.1, 7.0]	1.0 [1.0, 1.1]
Nutrition	540	65.2	34.2 [29.2, 39.2]*	2.1 [1.9, 2.3]*	530	90.9	13.8 [10.2, 17.5]*	1.2 [1.1, 1.2]*
N+WSH	555	63.8	33.1 [28.0, 38.2]*	2.1 [1.9, 2.3]*	517	91.1	13.8 [10.2, 17.4]*	1.2 [1.1, 1.2]*

a Prevalence differences (PD) were estimated using linear regression models adjusted for clustering comparing each intervention arm with the control; prevalence ratios (PR) were estimated using Poisson regression models adjusted for clustering.

b Statistical analysis was not performed due to a lack of variability in the outcome variables.

* *P* value < 0.05.

**Figure 2:**
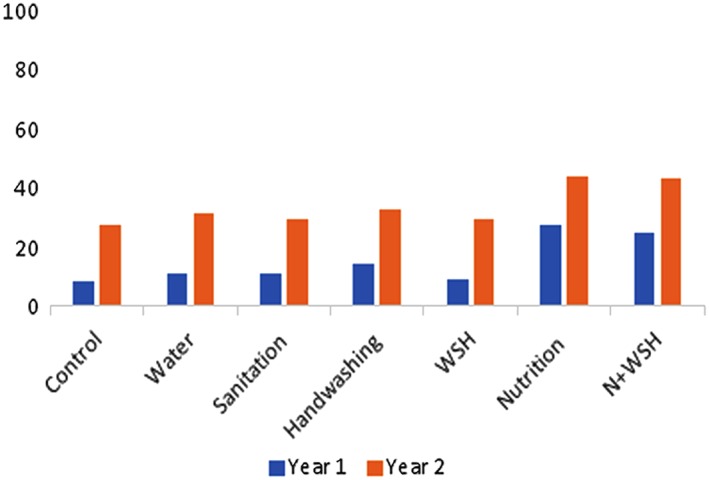
Proportion of children received ≥4 food groups every day in past 7 days

**Figure 3:**
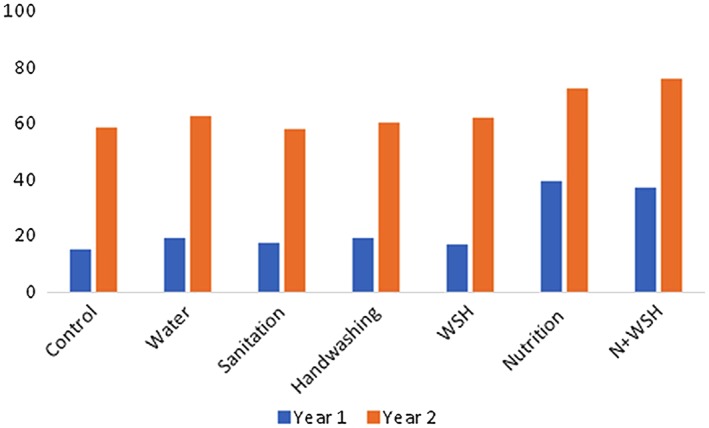
Proportion of children received animal source food every day in past 7 days

Using the 7‐day recall period, children in the nutrition intervention arms were also more likely to meet the MDD score for all days in the preceding 7 days compared with those in the control arm in both follow‐up periods (year 1: N: 27.7%; N+WSH: 25.0% vs. C:8.7%; year 2: N: 43.7%; N+WSH: 43.0% vs. C:27.8%). However, the overall proportion of meeting MDD score every day in the past 7 days was less than 50% (Figure 2). Similarly, children in the nutrition arms were more likely to receive animal source foods every day in past 7 days than children in the control arm (year 1: N: 39.1%; N+WSH: 37.1% vs. C:15.1%; year 2: N: 72.3%; N+WSH: 75.6% vs. C:58.1%) at both time points (Figure 3).

## 4 DISCUSSION

In this study of community‐based WASH and nutrition promotion interventions, we found that children from households that received the nutrition interventions were more likely to meet MDD and MAD criteria compared with the control arm over 2 years of follow‐up. This improvement in food consumption patterns was in addition to nutritional improvements achieved through the provision of LNS. Consumption of animal source food was more frequent among the nutrition intervention arms. CHWs' information about food groups and encouragement to provide family foods were effective in increasing dietary diversity for young children. The largest differences between arms were evident at the year 1 follow‐up. However, during the year 2 follow‐up visit, when the children were an average age of 22 months, the proportion meeting the MDD score among the non‐nutrition intervention arms and controls increased more than twofold from the year 1 measurement. This finding suggests that interventions targeting young children in their first year of life can be effective to encourage earlier introduction of diverse foods.

Household and community level behaviour change activities have been found crucial in improving complementary feeding practices in similar socio‐economic contexts including rural Zimbabwe and Ethiopia (Negash et al., 2014; Paul et al., 2012). A cluster‐randomized programme evaluation in Bangladesh (Menon et al., 2016) found that a combined intervention of intensive interpersonal counselling, mass media campaigns, and community mobilization substantially improved complementary feeding practices compared with a nonintensive programme. However, there was no difference in stunting rates between arms. A cluster‐randomized trial in rural India showed that responsive feeding education improved dietary intake and cognitive development of young children following 1 year of intervention; however, there was no impact on infant growth (Vazir et al., 2013). In our trial, we detected a significant improvement in child linear growth (Luby et al., 2018) in the two nutrition arms that received LNS together with counselling on improved infant and young child feeding practices.

There is limited evidence of advantages and challenges regarding integrated approaches that are optimal for children (Grantham‐McGregor, Fernald, Kagawa, & Walker, 2014). In this randomized control trial, we integrated WSH interventions together with a nutrition intervention package. Unlike some programmes that achieved differential uptake when multiple risk mitigation strategies were promoted (Cutler, 2004; Vazir et al., 2013), we found that the combined nutrition and WSH intervention was equally effective to the nutrition‐specific intervention approach at improving complementary feeding behaviour. Being an efficacy trial, the WASH Benefits Bangladesh study deployed an intensive intervention with frequent contact with the CHWs. Nevertheless, this resulted in a parallel intervention; the participants were equally likely to report improved complementary feeding and display evidence of improved WSH practices. Though the context and challenges were different than that of a large‐scale programme, WASH Benefits Bangladesh provided evidence of feasibility of integrated intervention including delivery, monitoring, and evaluation (Unicomb et al., 2018; Parvez et al., 2018; Rahman et al., 2018).

The consumption of food categories that were the most nutrient dense: legumes or nuts, egg, flesh foods, and vitamin‐A rich fruits and vegetables were most likely to have improved in the nutrition intervention arms. This finding suggests that LNS is unlikely to displace locally available healthy foods when their consumption is promoted in parallel with LNS. We found that an effective behaviour change intervention could improve overall diet quality in addition to improvements achieved via LNS supplementation. This result is consistent with other studies that have looked at the effect of LNS supplement distribution on complementary feeding practices in different populations such as Honduran children (Flax, Siega‐Riz, Reinhart, & Bentley, 2015) or Malawian infants (Hemsworth et al., 2016).

We conducted a clustered randomized controlled trial, which was rigorous in design and implementation. The study had statistical power to detect small effects. Randomization successfully generated balanced background characteristics across arms (Luby et al., 2018). Loss to follow‐up was around 16% over 2 years and balanced across arms. The trial was implemented with high fidelity, as evidenced by the high uptake of the WSH technologies and behaviours and LNS adherence in both the single and combined intervention arms (Parvez et al., 2018; Luby et al., 2018). On the other hand, this study suffered from some limitations. We did not measure the amount of food consumed by the children. Thus, it was not possible to quantify the total calorie, protein, or micronutrient intake. Complementary food consumption practices were reported by the mothers, and given that this study was not blinded to the participants or the data collectors, the data might have been affected by reporting bias. However, we used the standardized data collection methods and food group categories recommended by the WHO.

We conclude that household and community level behaviour change activities that aimed to optimize dietary diversity and feeding family foods were effective at improving dietary diversity during the study period among young children who participated in the study. Integration of WSH with the nutrition behaviours did not result in dilution of reported nutrition practices. Nutrition‐specific interventions and nutrition‐sensitive interventions such as WSH can be effectively combined to promote dietary diversity.

## CONFLICTS OF INTEREST

The authors declare that they have no conflicts of interest.

## CONTRIBUTIONS

KJ drafted the manuscript under the guidance of CPS and input from all listed co‐authors. SPL drafted the research protocol; he coordinated input from the study team throughout the project. PJW, EL, MR, LU, PKR developed the water, sanitation, and handwashing intervention and the overarching behaviour change strategy. CPS developed the nutrition intervention and guided the analysis and interpretation of these results. MR, LU, SMP, KJ, oversaw piloting and subsequent study implementation, contributed to refinements in interventions and measurements, and responded to threats to validity. KJ, KKD, CPS developed the analytical approach, conducted the statistical analysis, constructed the tables and figures, and helped interpret the results.

## Supporting information

Table S1: Effect of the intervention on infant and young child feeding practices comparing the combined nutrition arm (N+WSH) to the nutrition‐only arm

Table S2: Effect of the intervention on infant and young child feeding practices in multivariable adjusted models
