# Supplementary material for: Complementary feeding practices among rural Bangladeshi mothers: Results from WASH Benefits study
Source: Matern Child Nutr. 2018 Aug 13;15(1):e12654. doi: 10.1111/mcn.12654 (PMC6519265; doi:10.1111/mcn.12654)
Supplement: Supplementary file 1 — Table S1: Effect of the intervention on infant and young child feeding practices comparing the combined nutrition arm (N+WSH) to the nutrition‐only arm [file MCN-15-e12654-s001.docx]

| Table S1: Effect of the intervention on infant and young child feeding practices comparing the combined nutrition arm (N+WSH) to the nutrition-only arm | | | | | | | | | |
| --- | --- | --- | --- | --- | --- | --- | --- | --- | --- |
|  | **Year 1** | | | |  | **Year 2** | | | |
|  | N | % | PD^‡^ (95% CI) | PR (95%CI) |  | N | % | PD^‡^ (95% CI) | PR^‡^ (95%CI) |
| ***Minimum Dietary Diversity*** | |  |  |  |  |  |  |  |  |
| Nutrition | 548 | 66.4 | Ref | Ref |  | 574 | 91.5 | Ref | Ref |
| N+WSH | 563 | 65.0 | -1.1 (-7.1, 5.0) | 1.0 (0.9, 1.1) |  | 586 | 91.6 | 0.2 (-3.2, 3.5) | 1.0 (0.9, 1.0) |
| ***Minimum Meal Frequency*** | |  |  |  |  |  |  |  |  |
| Nutrition | 540 | 93.5 | Ref | Ref |  | 438 | 100 | Ref | Ref |
| N+WSH | 555 | 95.0 | 1.3 (-1.9, 4.4) | 1.0 (0.9, 1.0) |  | 427 | 99.8 | -0.2 (-0.6, 0.2) | 1 |
| ***Minimum Acceptable Diet*** | |  |  |  |  |  |  |  |  |
| Nutrition | 540 | 65.2 | Ref | Ref |  | 438 | 90.9 | Ref | Ref |
| N+WSH | 555 | 63.8 | -1.1 (-7.2, 5.0) | 1.0 (0.9, 1.1) |  | 427 | 91.1 | 0.4 (-3.6, 4.5) | 1.0 (0.9, 1.1) |
| ^‡^Prevalence Differences (PD) were estimated using linear regression models adjusted for clustering comparing each intervention arm to the control; Prevalence Ratios (PR) were estimated using Poisson regression models adjusted for clustering. | | | | | | | | | |
