# Supplementary material for: Complementary feeding practices among rural Bangladeshi mothers: Results from WASH Benefits study
Source: Matern Child Nutr. 2018 Aug 13;15(1):e12654. doi: 10.1111/mcn.12654 (PMC6519265; doi:10.1111/mcn.12654)
Supplement: Supplementary file 2 — Table S2: Effect of the intervention on infant and young child feeding practices in multivariable adjusted models [file MCN-15-e12654-s002.docx]

| Table S2: Effect of the intervention on infant and young child feeding practices in multivariable adjusted models | | | | | | | | | |
| --- | --- | --- | --- | --- | --- | --- | --- | --- | --- |
|  |  |  |  |  |  |  |  |  |  |
|  | **Year 1** | | | |  | **Year 2** | | | |
|  | N | % | PD^‡^ (95% CI) | PR^‡^ (95%CI) |  | N | % | PD^‡^ (95% CI) | PR^‡^ (95%CI) |
| ***Minimum Dietary Diversity*** | |  |  |  |  |  |  |  |  |
| Control | 1103 | 32.4 | Ref | Ref |  | 1138 | 77.7 | Ref | Ref |
| Water | 578 | 36.0 | 3.2 (-1.6, 8.0) | 1.1 (1, 1.3) |  | 598 | 80.8 | 2.8 (-1.2, 6.7) | 1.0 (1.0, 1,1) |
| Sanitation | 552 | 37.1 | 4.7 (0.3, 9.1) | 1.1 (1, 1.3) |  | 585 | 82.0 | 3.9 (0.1, 7.6) | 1.1 (1.0, 1.1) |
| Handwashing | 555 | 40.4 | 7.8 (3.1, 12.4)^*^ | 1.2 (1.1, 1.4)^*^ |  | 570 | 81.1 | 3.3 (-0.4, 7.0) | 1.0 (1.0, 1.1) |
| WSH | 570 | 36.5 | 3.7 (-0.5, 7.9) | 1.1 (1, 1.3) |  | 588 | 80.3 | 2.2 (-1.5, 6.0) | 1.0 (1.0, 1.1) |
| Nutrition | 548 | 66.4 | 34.3 (29.3, 39.3)^*^ | 2.1 (1.9, 2.3)^*^ |  | 574 | 91.5 | 13.8 (10.3, 17.2)^*^ | 1.2 (1.1, 1.2)^*^ |
| N+WSH | 563 | 65.0 | 33.1 (28.1, 38.1)^*^ | 2.0 (1.8, 2.3)^*^ |  | 586 | 91.6 | 13.9 (10.5, 17.2)^*^ | 1.2 (1.1, 1.2)^*^ |
| ***Minimum Meal Frequency*** | |  |  |  |  |  |  |  |  |
| Control | 1074 | 85.0 | Ref | Ref |  | 1020 | 100 | -^§^ | -^§^ |
| Water | 563 | 86.3 | 1.4 (-2.2, 5.0) | 1.0 (1.0, 1.1) |  | 532 | 100 | -^§^ | -^§^ |
| Sanitation | 539 | 87.8 | 3.1 (-0.1, 6.4) | 1.0 (1.0, 1.1) |  | 538 | 100 | -^§^ | -^§^ |
| Handwashing | 549 | 85.4 | 0.4 (-3.2, 4.1) | 1.0 (0.9, 1.0) |  | 518 | 100 | -^§^ | -^§^ |
| WSH | 553 | 88.3 | 3.5 (-0.1, 7.0) | 1.0 (1.0, 1.1) |  | 529 | 100 | -^§^ | -^§^ |
| Nutrition | 540 | 93.5 | 8.9 (5.8, 12.1) | 1.1 (1.1, 1.1) |  | 530 | 100 | -^§^ | -^§^ |
| N+WSH | 555 | 95.0 | 10.2 (7.2, 13.2) | 1.1 (1.1, 1.2) |  | 517 | 99.8 | -^§^ | -^§^ |
| ***Minimum Acceptable Diet*** | |  |  |  |  |  |  |  |  |
| Control | 1074 | 30.7 | Ref | Ref |  | 1020 | 77.3 | Ref | Ref |
| Water | 563 | 34.5 | 3.4 (-1.4, 8.2) | 1.1 (1.0, 1.3) |  | 532 | 80.1 | 2.8 (-1.5, 7.1) | 1.0 (1.0, 1.1) |
| Sanitation | 539 | 35.3 | 4.5 (0, 8.9) | 1.1 (1.0, 1.3) |  | 538 | 81.0 | 3.7 (-0.4, 7.7) | 1.0 (1.0, 1.1) |
| Handwashing | 549 | 39.0 | 7.9 (3.2, 12.5)^*^ | 1.3 (1.1, 1.4)^*^ |  | 518 | 81.3 | 4.2 (0.3, 8.1)^*^ | 1.1 (1.0, 1.1) |
| WSH | 553 | 35.1 | 3.9 (-0.3, 8.1) | 1.1 (1.0, 1.3) |  | 529 | 80.3 | 2.8 (-1.2, 6.9) | 1.0 (1.0, 1.1) |
| Nutrition | 540 | 65.2 | 34.6 (29.6, 39.5)^*^ | 2.1 (1.9, 2.4)^*^ |  | 530 | 90.9 | 13.9 (10.2, 17.6)^*^ | 1.2 (1.1, 1.2)^*^ |
| N+WSH | 555 | 63.8 | 33.2 (28.1, 38.3)^*^ | 2.1 (1.9, 2.3)^*^ |  | 517 | 91.1 | 13.8 (10.2, 17.4)^*^ | 1.2 (1.1, 1.2)^*^ |
| ^*^P-value <0.05  ^‡^Prevalence Differences (PD) were estimated using linear regression models adjusted for clustering comparing each intervention arm to the control; Prevalence Ratios (PR) were estimated using Poisson regression models adjusted for clustering. The models include covariates: child sex, father’s occupation, parent’s education, household income, geographic location/study site, and seasonality.  ^§^Statistical analysis was not performed due to a lack of variability in the outcome variables. | | | | | | | | | |
|  | | | |  |  |  |  |  |  |
